# Development of non-defective recombinant densovirus vectors for microRNA delivery in the invasive vector mosquito, *Aedes albopictus*

**DOI:** 10.1038/srep20979

**Published:** 2016-02-16

**Authors:** Peiwen Liu, Xiaocong Li, Jinbao Gu, Yunqiao Dong, Yan Liu, Puthiyakunnon Santhosh, Xiaoguang Chen

**Affiliations:** 1 Guangdong Provincial Key Laboratory of Tropical Disease Research, Department of Pathogen Biology, School of Public Health and Tropical Medicine, Southern Medical University, Guangzhou, Guangdong, 510515, China; 2 Reproductive Medical Center of Guangdong Women and Children Hospital, Guangzhou, Guangdong, 511442, China

## Abstract

We previously reported that mosquito densoviruses (MDVs) are potential vectors for delivering foreign nucleic acids into mosquito cells. However, considering existing expression strategies, recombinant viruses would inevitably become replication-defective viruses and lose their ability for secondary transmission. The packaging limitations of the virion represent a barrier for the development of MDVs for viral paratransgenesis or as high-efficiency bioinsecticides. Herein, we report the development of a non-defective recombinant Aedes aegypti densovirus (AaeDV) miRNA expression system, mediated by an artificial intron, using an intronic miRNA expression strategy. We demonstrated that this recombinant vector could be used to overexpress endogenous miRNAs or to decrease endogenous miRNAs by generating antisense sponges to explore the biological functions of miRNAs. In addition, the vector could express antisense-miRNAs to induce efficient gene silencing *in vivo* and *in vitro*. The recombinant virus effectively self-replicated and retained its secondary transmission ability, similar to the wild-type virus. The recombinant virus was also genetically stable. This study demonstrated the first construction of a non-defective recombinant MDV miRNA expression system, which represents a tool for the functional analysis of mosquito genes and lays the foundation for the application of viral paratransgenesis for dengue virus control.

Mosquito-borne diseases continue to pose a public health threat^1^. The Asian tiger mosquito, *Aedes albopictus*, is an aggressive, day-time biting insect that is emerging throughout the world as a public health threat following its primary role in recent dengue (DENV) and Chikungunya (CHIKV) outbreaks^2^,^3^. Chemical insecticides, which have traditionally been used in response to epidemics, are a major part of a sustainable, integrated mosquito management system for the prevention of mosquito-borne diseases. However, issues with current control strategies, such as the emergence of insecticide resistance and negative environmental impacts, necessitate the need for novel disease prevention measures^4^,^5^. Therefore, there is a concerted effort to develop novel strategies to combat arthropod-borne diseases.

Paratransgenesis is the genetic manipulation of vector endosymbiotic microorganisms, such as bacteria, viruses or fungi. Paratransgenesis has been proposed as a potential method to control vector-borne diseases, which act to inhibit either pathogen or vector development by manipulating essential coding RNA genes or small RNAs involved in vector development, reproduction and/or interactions between the host and the pathogen^6^,^7^,^8^,^9^. For mosquitoes, the genetic modification of bacteria and fungi has been shown to significantly inhibit pathogen levels in *Anopheles gambiae*^10^,^11^. However, concerns regarding how to effectively introduce engineered bacteria or fungi into mosquitoes in the field and how to avoid potential risks to non-target insects remain huge challenges for further applications.

Mosquito densoviruses (MDVs) are non-enveloped, single-stranded DNA viruses that belong to the genus *Brevidensovirus* of the subfamily *Densovirinae* in the family *Parvoviridae.* MDVs are relatively stable in the environment and have the potential to spread and persist naturally in mosquito populations by both horizontal and vertical transmission. Most importantly, MDV host specificity is apparently restricted to mosquitoes. MDVs have the potential for vector control as transducing agents to express foreign toxins or small interfering RNAs molecules *in vitro* and *in vivo*. However, regardless of what type of expression strategies are selected, recombinant viruses inevitably become replication-defective viruses and lose their ability for secondary transmission due to the removal of non-structural and/or the viral capsid protein (VP) genes, which are essential for viral packaging and replication.

Herein, we report the development of a non-defective recombinant Aedes aegypti densovirus (AaeDV) miRNA expression system mediated by an artificial intron. This recombinant vector can be used to not only overexpress the endogenous miRNAs or to decrease endogenous miRNAs by generating an antisense sponge for miRNAs biological functions exploration but also express amiRNAs to induce efficient gene silencing *in vivo* and *in vitro*. This study demonstrates the first construction of non-defective recombinant MDVs.

## Materials and Methods

### Mosquito cell maintenance and mosquito rearing

*Ae. albopictus* C6/36 cell lines (ATCC CRL-1660) were cultured at 28 °C in Roswell Park Memorial Institute (RPMI) 1640 medium (Gibco, Life Technology, China) supplemented with 10% foetal bovine serum (Gibco, Life Technology, Australia). The *Ae. albopictus* Foshan strain used in this work was obtained from the Guangdong Province, China and was established in the laboratory in 1981. Mosquitoes were reared at 28 °C with 70% to 80% humidity under a 12-h darkness/12-h light regime. Larvae were reared in pans and fed on finely ground fish food, mixed 1:1 with yeast powder. Adult mosquitoes were kept in 30-cm cube cages and allowed access to a cotton wick soaked in 20% sucrose as a carbohydrate source. Adult females were allowed to bloodfeed on anesthetized mice 3 and 4 days after eclosion. Each batch of mosquitoes was tested by conventional PCR to ensure that the experimental mosquitoes were free of MDVs (data not shown).

### Artificial intron, miRNA sponges and amiRNAs design

**Figure 1:**
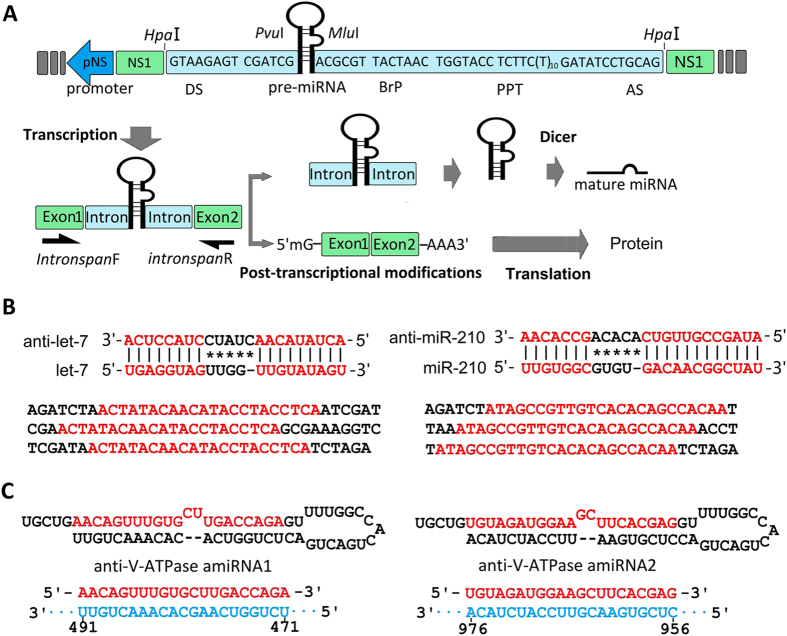
Biogenesis of artificial intronic microRNA (miRNA) and the strategy to generate the miRNA sponges and artificial miRNAs. (**A**) The artificial intron is shown flanked by a splice donor (DS) and an acceptor site (AS) and contains a branch-point domain (BrP), a poly-pyrimidine tract (PPT) and pre-miRNA. The miRNA sponge or artificial miRNA sequence is inserted inside the intron, located between the 5-splice site and the BrP. The intronic miRNA is co-transcribed within a precursor messenger RNA (pre-mRNA) of NS1 driven by the pNS1 promoter and cleaved out of the pre-mRNA by RNA splicing. Although the exons are ligated to form a mature messenger RNA (mRNA) for NS1 protein synthesis, the spliced intron with the pre-miRNA is further processed into mature miRNA by Dicer. (**B**) The strategy to generate the anti-let-7 and anti-miR-210 constructs. Alignment of anti-let-7 and anti-miR-210 sequences with aal-let-7 and aal-miR-210, respectively. Complete matching of the seed regions with the anti-miRNA sequence and tail regions are shown. Both let-7 and miR-210 sponges contain three repeat antisense constructs (red letters) that can bind to aal-let-7 and aal-miR-210, respectively. (**C**) Sequences and predicted precursor structures for the miRNA-based artificial miRNAs used in this study. The mature artificial miRNAs are shown in red, and their related target mRNA sequences are in blue. The target sequences locations are shown below.

The artificial intron used in this work was described previously^12^. The essential components of the artificial intron include several consensus nucleotide elements consisting of a 5′-splice site, a branch-point motif (BrP), a poly-pyrimidine tract (PPT), and a 3′-splice site (Fig. 1A). Endogenous precursor miRNAs of *Ae. albopictus* aal-let-7 and aal-mir-210 were selected to test the suitability of recombinant virus-based miRNA expression vectors for miRNAs overexpression. The precursors and mature sequences of aal-let-7 and aal-mir-210 were described previously (see also Supplementary Table S1)^13^.

To explore the ability of AaeDV as a virus-based miRNA suppression system (VbMS), the anti-miRNA sponges targeting endogenous aal-let-7 and aal-miR-210 were introduced into the AaeDV. Both anti-miRNA sponge constructs are shown in Fig. 1-B (see also Supplementary Table S1) and contained three repeat antisense sequences that completely matched the seed regions of the target miRNAs.

To verify the feasibility of AaeDV-based artificial microRNA-mediated gene silencing *in vivo* and *in vitro*, the *Ae. albopictus* vacuolar ATPases gene (*V-ATPase*; GenBank:AY864912) was selected as the target gene. Artificial miRNA sequences were designed using BLOCK- iT™ RNAi Designer (http://rnaidesigner.lifetechnologies.com/rnaiexpress/) (Fig. 1-C, Supplementary Table S1) and then subcloned into AaeDV. A human-specific miR-941-1 precursor^14^ and its sponge were also subcloned into AaeDV as a control.

All endogenous precursor miRNAs, antisense sponges and artificial miRNAs were inserted inside the artificial intron, located between the 5′-splice site and the branch-point motif (BrP) to generate intronic miRNA expression constructs, respectively. All the constructs were produced by chemical synthesis and then subcloned into the pJET1.2/blunt cloning vector using a CloneJET PCR Cloning Kit (Thermo Scientific, Life Technology, USA).

### Plasmid construction

**Figure 2:**
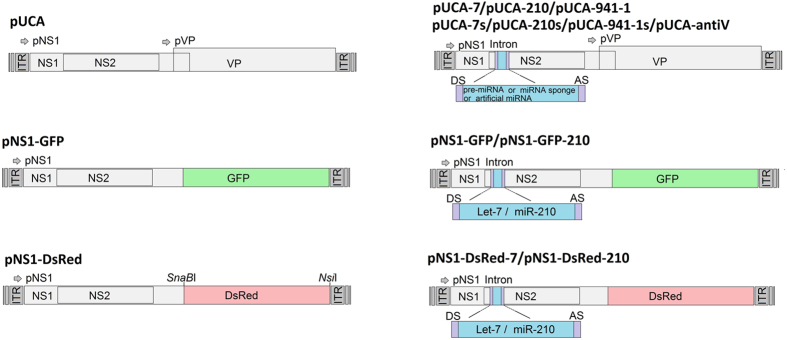
Schematic organization of the recombinant *Aedes aegypti* densovirus (AaeDV) plasmids. The pNS and pVP viral promoters drive the expression of the NS1 and NS2 genes and VP genes, respectively. In the plasmids p7NS1-GFP and p7NS1-DsRed, the GFP and DsRed gene were fused to the NS1 gene, respectively. pCUA-7, pUCA-210, pCUA-7s, pUCA-210s, pUCA-941-1, pUCA-941-1s and pUCA-antiV1/2, contain the artificial introns, including the aal-let-7, aal-miR-210, aal-let-7 sponge, aal-miR-210 sponge, hsa-miR-941-1, hsa-miR-941-1 sponge and artificial miRNA, respectively, which were cloned into the *Hpa*I site of the NS1 gene.

pUCA is an infectious clone containing the AaeDV genome (3,981 nt) in pUC19^12^. pUCA was kindly provided by Prof. Jonathan Carlson and has been previously described in detail^11^. p7NS1-GFP expresses an non-structural protein 1 (NS1)-green fluorescent protein (GFP) fusion protein from the p7 promoter. The construction of p7NS1-GFP has been described in detail elsewhere^11^. pNS1-DsRed was constructed by inserting a red fluorescent protein (DsRed) fragment was into the *SnaB*I*-Nsi*I site of pUCA. As a result, the DsRed was fused to the C-terminus of non-structural protein NS1, and the expression of the fusion protein gene was driven by the pNS1 promoter (Fig. 2).

All the intronic expression constructs, including aal-let-7, aal-mir-210, aal-let-7 sponge, aal-miR-210 sponge, two anti-*V-ATPase* amiRNA, hsa-mir-941-1 and hsa-miR-941-1 sponge were separately inserted into the *Hpa*I site in the AaeDV NS1 coding region using an In-fusion HD cloning kit (Clontech, CA, USA). The resulting plasmid constructs were called pUCA-7, pUCA-210, pUCA-7s, pUCA-210s, pUCA-antiV1, pUCA-antiV2, pUCA-941-1, and pUCA-941-1s. Intronic constructs aal-let-7 and aal-mir-210 were also inserted into same location of p7NS1-GFP and pNS1-DsRed and named p7NS1-GFP-7, p7NS1-GFP-210, pNS1-DsRed-7 and pNS1-DsRed-210. All plasmids were confirmed by direct sequencing. One Shot^®^ Stbl3™ Chemically Competent *Escherichia coli* (Invitrogen, Life Technologies, CA, USA) were used for all cloning procedures and plasmid preparation. The plasmids that were used in this study are shown in Fig. 2.

### Mosquito cell transfection and recombinant virus production

One day before transfection, 2 × 10^5^ cells per well were plated in six-well plates. The transfection of plasmids was performed using Lipofectamine 2,000 (Invitrogen), according to the manufacturer’s protocol. Supercoiled plasmids used for transfection were prepared using a GeneJET Endo-Free Plasmid Maxiprep Kit (Thermo Scientific, Life Technologies, CA, USA).

Recombinant viruses (VrepUCA-7, VrepUCA-210, VrepUCA-7s, VrepUCA-210s, VrepUCA-antiV1/2, VrepUCA941-1 and VrepUCA-941-1s) and control wild-type AaeDV were generated by transfecting the corresponding infection clones pUCA-7, pUCA-210, pUCA-7s, pUCA-210s, pUCA-antiV1/2, pUCA941-1, pUCA941-1s and pUCA into C6/36 cells, according to the manufacturer’s protocol. After a 5-day incubation, infected cells were harvested using cell scrapers, lysed by freezing and thawing, and then centrifuged for 10 min at 1,000 × *g*. The supernatants were kept as virus stocks. Defective virus VrepNS1-DsRed was used to express an NS1-DsRed fusion protein for *in vivo* tracing and was generated by cotransfecting pNS1-DsRed with helper plasmid pUCA-7, pUCA-210, pUCA-7s, pUCA-210s, pUCA941-1, pUCA941-1s or pUCA (the co-transfection concentration ratio was 2:1). Defective viruses VrepNS1-GFP-7, VrepNS1-GFP-210, VrepNS1-DsRed-7 and VrepNS1-DsRed-210 were used to detect the splicing of artificial intron *in vivo* and were generated by cotransfecting pNS1-GFP-7, pNS1-GFP-210, pNS1-DsRed-7 and pNS1-DsRed-210 with the helper plasmid pUCA (the co-transfection concentration ratio was 2:1). Recombinant virus production followed the method described above; the supernatants were kept as the tracer virus, and the recombinant virus was kept as mixed stocks.

### Mosquito transduction

1^st^ and 2^nd^ instar *Ae. albopictus* larvae were exposed to recombinant viruses VrepUCA-7, VrepUCA-210, VrepUCA-7s, VrepUCA-210s, VrepUCA-antiV1/2, VrepUCA941-1 and VrepUCA-941-1s as mixed stocks at a concentration of 1.00 × 10^10^ copies/ml by introducing them into a beaker containing 100 ml deionized water and 5 ml of the mixed virus stocks. AaeDV mixed stocks were used as negative controls. The blank control group, which received no virus, was exposed to C6/36 cell culture medium under identical conditions to the treatment groups. After incubation for 24 h at 28 °C, the larvae were transferred back to the pans and fed regularly. Once the fluorescent larvae were detected post-exposure, they were separated into an individual test plastic cup to allow subsequent continuous observation. Fluorescent signals were observed under an inverted fluorescence microscope, and photographs were captured using a Nikon ACT-2U digital camera (TE2000, Nikon, Tokyo, Japan). The data were processed and superimposed using Adobe Photoshop 7.0 software (Adobe Systems Inc., CA, USA).

### RT-PCR analysis of splicing efficiency of artificial introns

The total RNA was extracted from the C6/36 cells transfected with different intronic miRNA expression constructs at 96 h post transfection using the TRIzol reagent (Invitrogen). Any residual DNA was removed using a TURBO DNA-free™ Kit (Ambion, Life Technologies, TX, USA). First-strand cDNA was synthesized from the total RNA using Oligo (dT) primers and a RevertAid First Strand cDNA Synthesis Kit (Thermo scientific). Intron-spanning primers for the intronic miRNA expression constructs were designed. The mRNA of *Ae. albopictus* rpS7 (ribosomal protein 7) gene (GenBank: JN132168) was used as an internal control. All of the primers used in this study are shown in Supplementary Table S2.

### Quantitative real-time PCR (qPCR)

Total RNA was extracted from the C6/36 cells at 12 h, 24 h, 48 h, 72 h and 96 h post-transfection, and mosquito larvae at 5 d post-recombinant virus transduction. The mature miRNAs were quantified via qPCR using an SYBR Green I assay. The total RNA was purified using TRIzol reagent, and miRNAs were reverse transcribed using the miRcute miRNA First-Strand cDNA Synthesis Kit (Tiangen Biotech, Beijing, China) according to the manufacturer’s instructions. The reference gene was *Ae. albopictus* 5S ribosomal RNA (5S rRNA) (GenBank: L22060). The relative expression levels of the miRNAs were calculated using the comparative cycle threshold (Ct) method. The fold changes in miRNAs were calculated according to the equation 2^−ΔΔCt^.

For the quantitative mRNA analysis, an RNA extraction and a cDNA synthesis were used according to the procedures described above. The relative expression level of *V-ATPase* mRNA was normalized to rpS7 mRNA. Reactions were performed using a Super Real PreMix Plus (SYBR Green) (Tiangen Biotech). Each sample was assessed in triplicate. The qPCR results were analysed using the 2^−ΔΔCT^ method. The genome copy numbers of the recombinant virus and AaeDV were also quantified via qPCR, as described previously^15^. Non-encapsidated genome DNA and plasmid DNAs in the mix stocks were removed by TURBO DNase (Ambion, Life Technologies, USA). The total encapsidated genome DNA was extracted using a MiniBEST Viral RNA/DNA Extraction Kit Ver.5.0 (Takara, Japan). A standard curve was constructed by making serial 10-fold dilutions of a linear plasmid at known concentrations. Details of these procedures were described previously. The primers for aal-miR-210, aal-let-7, 5SrRNA, *V-ATPase*, rpS7 and virus copy numbers are shown in Supplementary Table S2.

### Purification of recombinant densovirus vectors particles and TEM transmission electron microscopy

Recombinant virus-infected C6/36 cells were harvested using cell scrapers, lysed by freezing and thawing, and then centrifuged for at 10,000 × *g* for 30 min to remove the cell debris. The supernatant was filtered using 0.22-μm filters and then centrifuged at 35,000 rpm for 75 minutes at 4 °C to pellet virion particles. The virion pellet was removed and further purified by 1 M sucrose cushion centrifugation for 120 minutes at 39,000 rpm, 4 °C. The final pellet was fractionated in a CsCl (0.3 g/ml) gradient at 60,000 rpm overnight at 8 °C. The virion band was removed from the gradient for DNA extraction and TEM. Purified virus particles were applied to glow-discharged carbon-coated grids and negatively stained with 2% (w/v) uranyl acetate. Electron micrographs were recorded on Kodak SO-163 film using a Philips CM12 electron microscope at nominal magnifications of 37,000× to 52,000×.

### Statistical analysis

Significant differences among the data groups were analysed in GraphPad Prism 6 using an unpaired t-test. P values were set at 0.05 (*p* < 0.05) for significant differences (*), 0.01 (*p *< 0.01) for highly significant differences (**), and 0.001/0.0001 (*p* < 0.001/*p*<0.0001) for extremely significant differences (***/****). Virus increases over time were significant based on an ANOVA test.

## Results

### The intronic miRNA expression constructs were effectively spliced into mosquito cells

We inserted the pre-miRNAs and miRNA sponges into an artificial intron between the 5′-splice site and BrP to generate intronic miRNA expression constructs. Theoretically, the intron would be co-transcribed with the precursor mRNA (pre-mRNA) of the recombinant virus NS1 gene driven by the pNS promoter and cleaved out of the pre-mRNA by RNA splicing. Subsequently, the spliced out intronic miRNA expression constructs, as 5′- and 3′-tailed mirtrons, generate the pre-miRNA hairpin with additional ribonucleolytic processing^16^, and then the pre-miRNA are further processed by the RNase Dicer into mature miRNAs. The exons of the NS1 gene transcript are then linked together to form a mature mRNA molecule for translational synthesis of entire NS1 protein.

**Figure 3:**
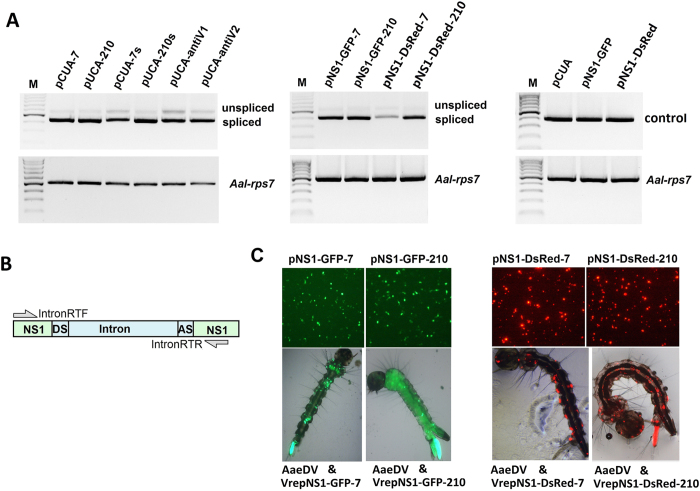
Splicing analysis of the intronic miRNA expression constructs in mosquito. (**A**) The horizontal arrows indicate the positions of the primers used for the expression splicing analysis of the intronic miRNA expression constructs. Amplification with IntronRTF and IntronRTR revealed a 407-bp major band that corresponded to ligation of the NS1 exon, whereas the 540-bp faint band corresponded to the non-spliced transcript. Amplification of rpS7 was used as a loading control. Visible bands were gel purified and sequenced. (**B**) Plasmids pNS1-GFP-7, pNS1-GFP-210, pNS1-DsRed-7 and pNS1-DsRed-7 transfected into C6/36 cells are shown in the top panel. Recombinant virus VrepNS1-GFP-7, VrepNS1-GFP-210, VrepNS1-DsRed-7 and VrepNS1-DsRed-7 transduced larvae are shown in the bottom panel. If the artificial intron is not removed, the frame-shift mutation in the fused fluorescence reporter will preclude the fluorescence. We detected fluorescence in all of the vector-transfected cells and in the recombinant virus-transduced larvae, confirming that the artificial intron functioned in the mosquito cells.

The artificial intron displayed effective splicing out of the precursor messenger RNA in mammalian cells in a previous study^12^. To trace whether the artificial intron could be effective removed during pre-mRNA splicing in mosquito cells, we performed an *in vitro* analysis of pre-mRNAs using RT–PCR with primers designed to amplify sequences spanning the intron. In all the artificial intron-inserted recombinant virus transfected cells, amplification with IntronRTF and IntronRTR primers using cDNA template synthesized from mature mRNAs with oligo(dT) revealed a 407-bp major band that corresponded to ligation of the NS1 exon, whereas the 540-bp faint band was a non-spliced transcript (Fig. 3). Moreover, the fluorescence of GFP and DsRed also provided robust markers for effective splicing of the artificial intron into the mosquito cells. For plasmids pNS1-GFP-7, pNS1-GFP-210, pNS1-DsRed-7 and pNS1-DsRed-7, if the artificial intron is not removed, the frame-shift mutation in the fused GFP marker will preclude fluorescence. We detected fluorescence in all of the vector-transfected cells and in the recombinant virus transduced larvae, confirming that the artificial intron was functional in the mosquito cells (Fig. 3).

### MDVs vector can be used to overexpress endogenous miRNAs

**Figure 4:**
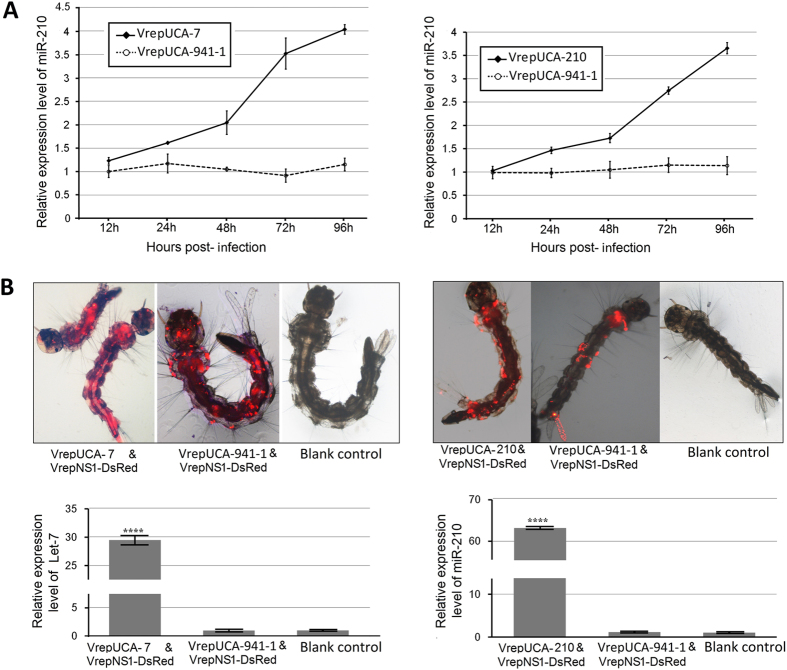
Recombinant MDVs-mediated endogenous miRNA overexpression *in vitro* and *in vivo*. (**A**) Analysis of endogenous miRNA mRNA expression in C6/36 cells after infection with the recombinant virus. (**B**) The larvae with DsRed expression that were distributed throughout the body were selected for the miRNA expression level quantitative assessment (top panel). Analysis of endogenous miRNA expression in larvae post-transduced with the recombinant virus. miRNA abundance was normalized to 5S rRNA, and the data are displayed as the mean ± SD of three biological replicates where an asterisk indicates significance between infected and corresponding mock treatments. A t-test was performed at different levels of significance. The asterisks represent statistically significant differences from the control (four asterisks, *p* < 0.0001).

Overexpression and suppression of miRNAs are the most widely used approaches to study miRNA function^17^. To determine whether AaeDV-based vectors are suitable for studying the function of endogenous mosquito miRNAs, we used the AaeDV vector to express miR-210 and let-7 of *Ae. albopictus*, respectively, *in vitro* and *in vivo.* VrepUCA-941-1 expressing human-specific microRNA miR-941-1was used as the negative control, and uninfected cells were used as a blank control. As shown in Fig. 4-A, cells infected with VrepUCA-7 exhibited overexpression of let-7 at 125.74 ± 4.40% (*p* = 0.014) compared to the negative control at 12 h post-infection, reaching a peak at 96 h (409.69 ± 1.19%) (*p* = 1.95 × 10^−5^). The VrepUCA-210 infected group exhibited significant overexpression of miR-210 at 149.61 ± 4.60% (*p* = 0.001) compared to the control at 24 h post-infection and reached its highest level at 96 h (323.14 ± 13.55%) (*p* = 4.29 × 10^−5^). Defective virus VrepNS1-DsRed was co-transduced as a tracer with the recombinant viruses in larvae, and 10 larvae with DsRed expression distributed throughout their bodies were selected (Fig. 4-B top panel) as a group for miRNA expression level quantification. Mosquito larvae infected with VrepUCA-7 and VrepUCA-210 exhibited overexpression of let-7 and miR-210 at 3639.04 ± 215.98% (*p* = 0.0006) and 7202.26 ± 250.34% (*p* = 0.0003) compared to the negative control (Fig. 4-B bottom panel). In contrast, no significant expression changes were observed in the negative control group VrepUCA-941-1 compared with the blank control in the cells and larvae (*p* > 0.05).

### MDVs vectors could be used to generate an antisense sponge construct to decrease endogenous miRNA expression

**Figure 5:**
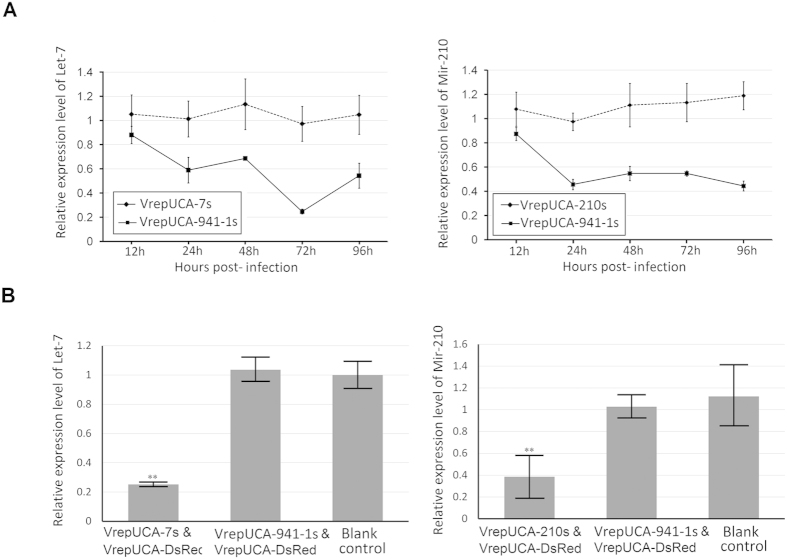
Recombinant virus-mediated microRNA sponge delivery for microRNA inhibition *in vitro* and *in vivo*. (**A**) The efficiencies of the anti-let-7 construct and anti-miR-210 construct in the C6/36 cells were tested. The constructs were able to decrease the levels of mature miRNAs of the let-7 and miR-210, as determined by real-time PCR. Error bars, SD (n = 3). (**B**) The efficiency of the anti-let-7 construct, and anti-miR-210 was detected in larvae. The construct downregulated the mature miRNAs of the let-7 andmiR-210 from the basal levels observed in the control cells, as determined via real-time PCR. Asterisks indicate that the differences reached the 0.05 significance level, and two asterisks indicate the 0.001 significance level.

A miRNA sponge was initially developed by Ebert and colleagues to inhibit miRNA function^18^. To study the feasibility of AaeDV-based vectors expressing an antisense sponge construct to decreases endogenous miRNA expression, we generated the constructs anti-let-7 and anti-miR-210, comprising three linked binding sites for let-7 or miR-210, which functioned as sponges to decoy and decay let-7 and miR-210 *in vivo* and *in vitro.* Anti- hsa-miR-941 constructs was used as a negative control, and uninfected cell served as the blank control. As shown in Fig. 5-A, C6/36 cells infected with VrepUCA-7s exhibited an inhibition ratio of 43.10 ± 10.22% (*p* = 0.008) compared to the control at 24 h post-transfection and reached a peak at 72 h (71.83 ± 2.15%) (*p* = 0.007), whereas pUCA-210s transfected cells displayed a stable inhibition ratio of approximately 50% (*p* < 0.05) at 24 post-infection. Recombinant virus infected larvae were isolated as described above for the *in vivo* assays. In VrepUCA-7s- and VrepUCA-210s-infected mosquito larvae, the inhibitions of let-7 and miR-210 were 78.46 ± 8.57% (*p* = 0.002) and 64.28 ± 9.06% (*p* = 0.004) compared to the negative control. There were no statistically significant differences between the blank control groups and the negative control groups for both the cells and larvae (*p* > 0.05) (Fig. 5-B).

### Impact of recombinant viruses-mediated miRNA over-expression and down-regulation of *Ae. albopictus* development

To estimate the impact of recombinant virus-mediated miRNA over-expression or down-regulation of *Ae. albopictus* development including larva stage span and pupation and emergence rates, *Ae. albopictus* larvae were exposed to concentrations of 1.00 × 10^8^ geq/ml of the VrepUCA-7, VrepUCA-210, VrepUCA-941-1, VrepUCA-7s, VrepUCA-210s and VrepUCA-941-1s. Each experiment was repeated three times. The larva stage span of these virus-infected larvae was delayed significantly compared to control larvae (*p* ≤ 0.05). In particular, the larval stage in VrepUCA-7s-infected group displayed a longer period compared to the other groups (*p* ≤ 0.05). However, the pupation and emergence rates of infected larvae showed no significant difference among all of the virus-infected groups (Table 1).

### The recombinant viruses were not defective

**Figure 6:**
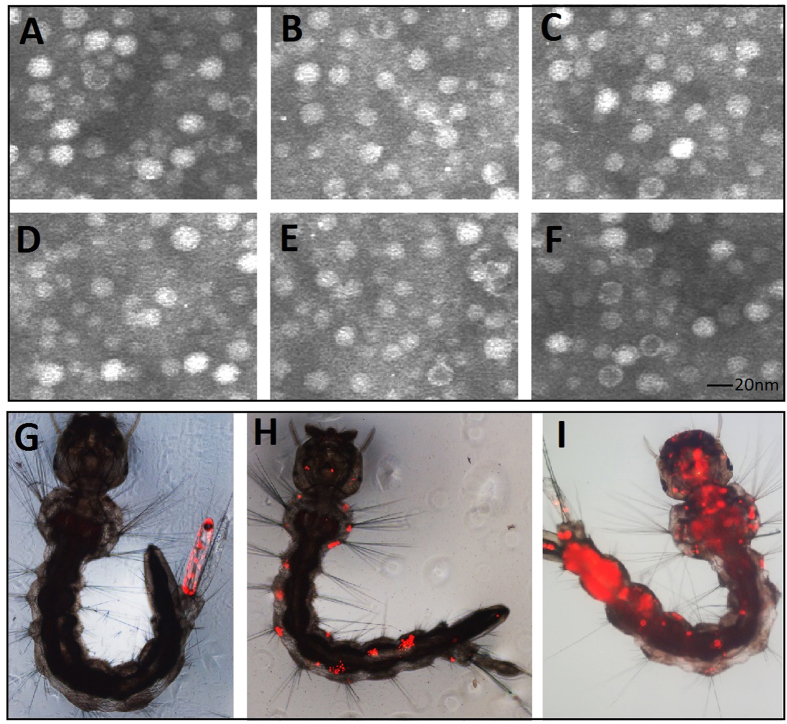
Electron microscopy of the recombinant virus particles from media of infected cultures. (**A–F**) Electron micrographs of the virus particles stained with 1% uranyl acetate. All of the recombinant viruses and wild-type viral particles displayed a homogeneous size (approximately 20 nm in diameter). (**A**) VrepUCA-7, (**B**) VrepUCA-210, (**C**) VrepUCA-7s, (**D**) VrepUCA-210s, (**E**) VrepUCA-antiV, and (**F**) AaeDV (recombinant Aedes aegypti densovirus). Primary infection site in Aedes albopictus larvae transduced with red fluorescent protein (DsRed) marker; (**G**) anal papillae; (**H**) bristle cell; (**I**) the whole body distribution of AaeDV from the primary infection site.

Recombinant viruses were produced as described in the materials and methods. To confirm that recombinant viruses VrepUCA-7, VrepUCA-210, VrepUCA-7s, VrepUCA-210s, and VrepUCA-antiV1 were not defective viruses, C6/36 cell were infected with the recombinant viruses. The old culture medium was removed completely, and 10 ml serum-free RPMI-1640 was added to wash the cells three times at 5 days post-transfection. We fractionated crude VrepUCA-7, VrepUCA-210, VrepUCA-7s, VrepUCA-210s and VrepUCA-antiV1 transfected C6/36 cell lysates in a caesium chloride gradient to purify the intracellular viral particles and examined them using negative-staining transmission electron microscopy. The results revealed numerous icosahedral, non-enveloped particles of the expected size (20 nm) (Fig. 6).

To compare the infection ability of the recombinant viruses with wild-type AaeDV for larvae, 20 new hatched larvae in each group were transduced with VrepUCA-7, VrepUCA-210, VrepUCA-7s, VrepUCA-210s, VrepUCA-antiV1 and AaeDV at a concentration of 1.00 × 10^10^ copies/ml. The genome DNA of every larva were isolated independently to detect the larval infection with virus specific primers 48 h post-infection using a PCR-based method, according to methods described previously^19^. Triplicate experiments showed that the larval infection rates of VrepUCA-7, VrepUCA-210, VrepUCA-7s, VrepUCA-210s and VrepUCA-antiV1 were 78.33 ± 5.77%, 85.00 ± 5.00%, 78.33 ± 7.64%, 80.00 ± 5.00% and 81.67 ± 7.64%, respectively. There was no significant difference between the recombinant viruses and AaeDV. To test the portal of entry and tissue tropisms of recombinant virus in larvae, we used the recombinant viruses VrepUCA-7, VrepUCA-210, VrepUCA-7s, VrepUCA-210s and VrepUCA-antiV1 to cotransduce larvae with the tracer VrepNS1-DsRed, and AaeDV served as a control. The DsRed marker expression was examined via fluorescence microscopy. Six groups of 500 new emerging larvae in each group were exposed to the recombinant virus mix stocks. The fluorescent larvae were separated continuously from 1 to 3 d post-exposure to the transducing particles.

The results revealed more than 80% of the larvae expressed GFP in each group (Supplementary Table S3). The major primary infection site was thought to be the anal papilla, followed by bristle cells, and finally the anal papilla. There were no significant differences between the recombinant virus and AaeDV. Investigation into the dissemination of the recombinant viruses in separated individual mosquitoes was based on daily monitoring of DsRed expression. The results showed that 54.60%, 58.40%, 59.40%, 54.60%, 52.20% and 54.80% of the infected larvae in the VrepUCA-7, VrepUCA-210, VrepUCA-7s, VrepUCA-210s, VrepUCA-antiV1 and AaeDV groups exhibited dissemination of the signal from the primary infection site to adjacent tissues and then to other tissues. As defective virions, VrepNS1-DsRed would not be generated independently by the defective genome and would lose the ability for the secondary transmission that takes place *in vivo* in mosquitoes unless a helper virus supplied the essential viral VP proteins. Therefore, both the icosahedral, non-enveloped recombinant virions and the defective VrepNS1-DsRed secondary transmission in larvae confirmed that the recombinant viruses were not defective.

### Stable propagation of the recombinant virus infectious clones in the bacterial host

**Figure 7:**
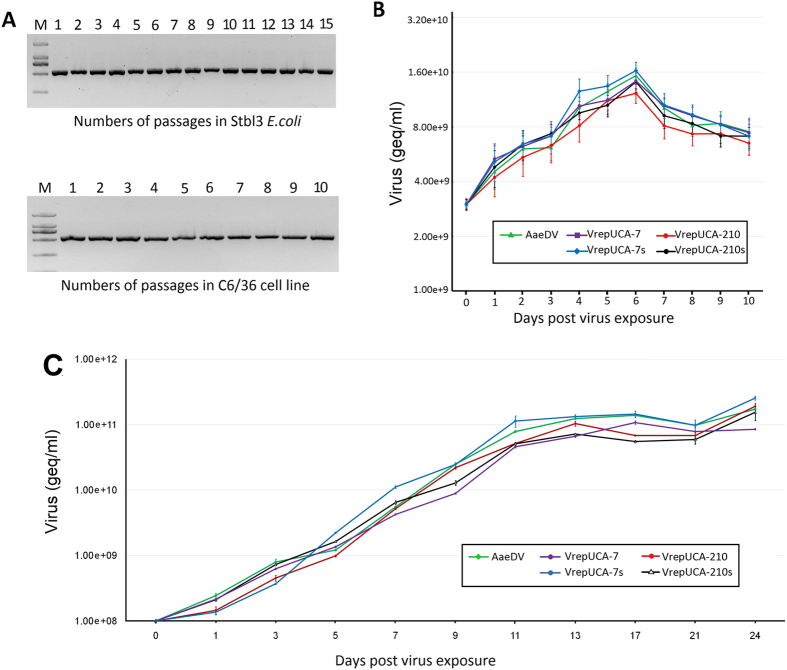
Viral genetic stability and proliferation studies of the recombinant virus. (**A**) Electrophoretic analysis of amplicons containing the insertion region obtained via PCR of the viral infectious clone pUCA-7 in Stbl3 *E. coli* cells (top panel). Analysis of the insert stability via genomic PCR of the viral RNA extracted from the supernatant of cultures after serial passages in the C6/36 cells (bottom panel). The number of passages is indicated above the figure. (**B**) Viral growth curves of the C6/36 cells. The cells were infected with either the control AaeDV or the recombinant VrepUCA-7, VrepUCA-210, VrepUCA-7s or VrepUCA-210s at 3.00 × 10^9^ geq/ml. Each time point represents the average titre obtained from three independent experiments with the respective standard deviations. (**C**) Accumulation of densovirus in the larval rearing water. Second instar larvae were exposed to 10^8^ geq/ml of VrepUCA-7, VrepUCA-210, VrepUCA-7s, VrepUCA-210 and AaeDV. The samples were analysed via real-time PCR and are reported as geq/ml. All viruses were produced in the C6/36 cells. Virus increase over time was significant based on an ANOVA test.

The infectious clones pUCA-7, pUCA-210 and pUCA-antiV were analysed for the genetic stability of their genome DNA to determine the suitability of the recombinant vectors for maintaining full-length genomic DNA. *E. coli* Stbl3 cells containing the infectious clones were passaged 15 times via overnight growth. The amplicons containing the insertion region were obtained via PCR amplification of the viral infectious clones in the Stbl3 *E. coli* cells and detected via gel electrophoretic analysis (Figs 7-A). Furthermore, the complete viral genome within the plasmid was sequenced after the 1st and 15th passages. No mutations were observed within the genome sequences after this extensive propagation in the bacterial host, indicating a highly stable system for the maintenance of complete genome sequences.

### Genetic stability of the viruses rescued from the infectious clones

To assess the genetic stability of VrepUCA-7, VrepUCA-210 and VrepUCA-antiV, we analysed amplicons containing the inserted artificial intron construct from viral genome samples after up to 10 serial passages in C6/36 cells. In all of the samples, the complete amplicon size of approximately 140 bp was detected, without any sign of continuous deletions in the heterologous cassette to up to 10 serial passages (Fig. 7-A). The high genetic stability was further confirmed by sequencing the complete VrepUCA-7, VrepUCA-210, VrepNS1-GFP-7, VrepNS1-GFP-210, VrepNS1-DsRed-7 and VrepNS1-DsRed-7 genomes from the 10th serial passage samples. We detected no point mutations in the heterologous gene or in the recombinant virus genome, indicating that this new recombinant virus is stable.

### Growth property of the recombinant virus

The proliferation capability of the VrepUCA-7, VrepUCA-210, VrepUCA-7s, VrepUCA-210s and AaeDV *in vivo* and *in vitro* were determined by absolute copy number of a densovirus gene using the SYBR Green PCR assay. In C6/36 cells, copy numbers of viruses were detected every day from day 1 up to day 10 post infection. In general, did not lead to an obvious decrease in recombinant virus yields compared to the w.t. AaeDV (*p* > 0.05) (Fig. 7-A).

The proliferation capability of the recombinant virus in larvae rearing water was also detected using the method described above. The equivalent gene copies of the recombinant virus VrepUCA-7, VrepUCA-210, VrepUCA-7s, VrepUCA-210s and AaeDV (2.00 × 10^10^ geq) were added to 200 ml of water containing 200 2^nd^ instar larvae. Samples of the larval-rearing water were collected during larval growth and analysed via real-time PCR to investigate whether the viruses were shed by the infected larvae and accumulated in the larval environment to facilitate horizontal transmission. The rearing water for the larvae exposed to all of the viruses after 1 d exhibited a significant increase in virus concentration (*p* < 0.05) (Fig. 7-C).

### Knockdown of V-ATPase *in vivo* and *in vitro*

**Figure 8:**
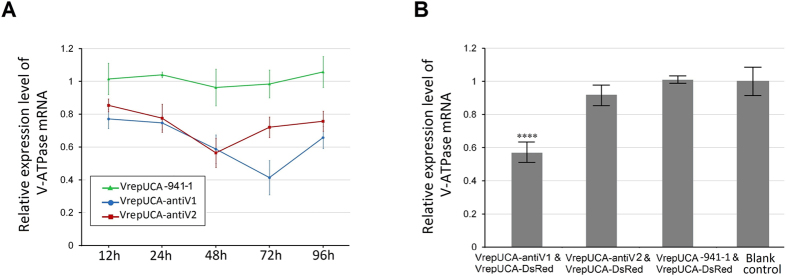
Recombinant virus-based artificial microRNA-mediated gene silencing *in vitr*o and *in vivo*. (**A**) Analysis of *V-ATPase* mRNA expression in the C6/36 cells after infection with the recombinant virus. (**B**) Detection of *V-ATPase* mRNA expression in *Ae. albopictus* larvae infected with the recombinant virus. Error bars represent the standard deviation of the 2^−ΔΔCT^ values for *V-ATPase* mRNA expression in the C6/36 cell line as evaluated via real-time RT-PCR.

To validate the effect of the artificial miRNA-mediated *V-ATPase* silencing, C6/36 cells were transfected with pUCA-antiV1/2, and the expression of *V-ATPase* was detected via real-time PCR at 12, 24, 48, 72 and 96 h post-transfection. The results showed that pUCA-antiV1 and pUCA-antiV2 exhibited significant silencing effects at all times points. The inhibition ratio of pUCA-antiV1 reached a peak at 72 h (57.16 ± 10.64%) (*p* = 0.003) and then declined. pUCA-antiV2 showed a relatively lower inhibition ratio than pUCA-antiV1 and reached a maximum at 48 h (39.94 ± 19.67%) (*p* = 0.019) (Fig. 8A).

*V-ATPase* silencing *in vivo* was investigated by cotransducing the recombinant viruses VrepUCA-7, VrepUCA-210 and VrepUCA-antiV into larvae with tracer VrepNS1-DsRed. Triplicate experiments showed that the VrepUCA-antiV1 and VrepUCA-antiV2 caused reductions in *V-ATPase* gene expression of 43.99 ± 2.90% (*p* = 0.0002) and 9.08 ± 4.75% (*p* = 0.036) compared to the control (Fig. 8B).

## Discussion

Many attempts have been made to manipulate MDVs as a favourable delivery vector in mosquitoes due to their characteristics of high host specificity, low cost, convenient administration, and horizontal transmission in the insect population. However, the limited packaging capacity of MDVs precludes the design of vectors associated with larger genes. The insert size testing suggests that 4,100–4,400 bp is the optimal genome size for packaging. The essential MDV inverted terminal repeats (ITRs), 3′ and 5′ untranslated regions as packing signals, promoter, polyadenylation, and enhancer sequences leave little or no space for most larger genes. MDVs have been reported that could be used as vectors to express the appropriately sized foreign gene, such as scorpion insect-specific neurotoxins^20^ and the GFP protein. However, wherever ORFs of MDV were inserted or replaced by foreign genes, the virions would not be generated independently due to the defective genome, unless a helper plasmid was cotransfected to supply the absent essential viral proteins. If recombinant MDVs are to be developed as paratransgenesis or bio-insecticide agents, the biological characteristics of vertical and horizontal transmission in nature must be preserved because a virus with a defective genome loses the ability for secondary transmission *in vivo*. To solve this problem, we used the intronic miRNA expression strategy.

Intronic miRNAs represent a new class of small single-stranded regulatory RNAs derived from introns that are co-transcribed within a precursor messenger RNA (pre-mRNA) by eukaryotic type-II RNA polymerases (Pol-II). The spliced intron then serves as a pri-miRNA for processing into an intronic precursor miRNA (pre-miRNA) or a multi-pre-miRNA cluster, which is then exported to the cytoplasm for final processing into mature miRNA by a miRNA-associated Dicer. An artificial intron-mediated miRNA expression system was reported by David TS Wu^21^, which contained synthetic RNA splicing and processing elements, such as the 5′-splice site, BrP, PPT and the 3′-splice site, to form an artificial intron containing a small hairpin RNA (shRNA) and an artificial miRNA. The construct effectively silenced their respective targeted genes in mammalian cells through an RNA interference (RNAi)-like mechanism.

An intronic miRNA expression cassette of no more than 200 nt can be introduced into the AaeDV genome without replacing any coding gene. The length of the recombinant virus genome is still suitable for packaging. In addition, the inserted artificial intron does not influence the ability of the exons to form a mature mRNA for protein synthesis, which indicates that virus self-replication and transmission are not disabled. Consequently, artificial introns spanning RT-PCR revealed that intronic miRNA cassettes were effectively spliced out from the pre-mRNA. Moreover, the fluorescence detection of constructs with DsRed and GFP markers in C6/36 cells also provided robust evidence that the artificial intron is not spliced (in this case, the frame-shift mutation precludes GFP or DsRed marker fluorescence). Both results confirmed that the artificial intron worked well in mosquito cells and did not influence the ligation of the inserted exon.

The major approaches used to study the functions of miRNAs include overexpression and loss-of-function. miRNA sponges are valuable tools for miRNA loss-of-function studies, both *in vitro* and *in vivo*, and encode a transcript composed of repeated miRNA antisense sequences that can sequester miRNAs from their endogenous targets. In insects, let-7 has been implicated in developmental timing^17^,^22^, wing development^23^,^24^ and neurogenesis^25^, and it is also associated with innate immunity by targeting the antimicrobial peptide diptericin^26^. miR-210, a conserved miRNA in insects, has been proved to be associated with learning and memory processes in mushroom bodies and the antennal lobes^27^,^28^ (among other places), and it is associated with pathogen infection as well^29^. Both let-7 and miR-210 displayed relatively low expression levels in *Ae. albopictus* larvae. In the present study, we demonstrated that a viral miRNA expression system can be used to effectively express endogenous miRNA, miRNA sponge and amiRNAs as a tool to study functions of endogenous miRNA genes. Furthermore, our results also displayed that endogenous let-7 down-regulation prolonged the development of *Ae. albopictus* larvae. Similar roles of let-7 in the control of developmental timing were also identified in *Drosophila melanogaster* and *Bombyx mori*, in which let-7 can regulate molting and metamorphosis by targeting key genes in the ecdysone pathway, and decay of let-7 caused arrestment during the molting^30^,^31^,^32^. Our results also suggest that let-7 was involved in the regulation of larval growth in *Ae. albopictus*. Similar developmental profiling of miR-210 was found in *Plutella xylostella*, *Anopheles stephensi* and *D. melanogaster*^13^,^33^,^34^,^35^, whereas decay of miR-210 did not show phenotypic effect on *Ae. albopictus* larvae. As reported previously, miR-210 played an important role in host microorganism interactions in adult mosquitoes^36^, so this miRNA seems unlikely to regulate the development in larval stage of *Ae. albopictus*.

Artificial microRNAs (amiRNAs) are 21-mer small RNAs that can be genetically engineered and function to specifically silence single or multiple genes of interest. In fact, the technique of RNA interference (RNAi) has a great potential for successful mitigation of various crop pest insects. For example, transgenic plants expressing amiRNAs targeting two different sites in the MpAChE2 gene exhibited better aphid resistance than the plants expressing MpAChE2-specific hairpin RNAs. V-ATPases comprise multimeric protein complexes and are commonly found in eukaryotic cell plasma membranes and the membranes of intracellular compartments^33^,^34^,^35^. *V-ATPases* are relatively conserved among species from bacteria to humans, particularly in mosquitoes^37^,^38^,^39^. Moreover, targeting the *V-ATPase* of insects is a promising approach in the fight against insect pests^37^,^38^. *V-ATPases* are essential for entry by many enveloped viruses and invasion into, or escape from, host cells by intracellular parasites^39^. RNAi-mediated silencing of several *V-ATPase* subunit genes results in reduced DENV infection^40^. Therefore, given its essential role in vector development and the interaction between host and pathogen, *V-ATPase* has become the preferred target gene for pest control and/or disruption of arbovirus transmission.

Infectious cloning is a powerful method for gene manipulation in the viral genome. Unfortunately, the instability of full-length viral cDNA or DNA in *E. coli* has been a major hurdle^41^,^42^,^43^,^44^,^45^. In particular, inverted repeat sequences (IRs) of viruses are considered ‘hot spots’ of genomic instability. Cloning of these DNA sequences in standard *E. coli* cloning hosts (such as DH5α or TOP10) led to plasmid instability due to deletions, mutations, duplications and rearrangements during bacterial propagation^46^, which is presumably caused by homologous recombination events. S strains of *E. coli*, such as Stbl3, have been developed to overcome some of these problems. These strains show reduced recombination of cloned DNA and are recommended for cloning of direct repeats and inverted repeat sequences suitable for blue/white screening with vectors capable of alpha-complementation. It is unclear whether the terminal inverted repeats of the recombinant MDVs have an effect on the cloning and propagation of the viral genome as a plasmid in *E. coli*. Therefore, we compared the stability of the recombinant virus with the wild-type AaeDV in *E. coli* Stbl3 passaged 10 times. No deletions, mutations or rearrangements were observed in the viral genomes of the wild-type or the recombinant or in the inserted foreign sequences, indicating a highly stable recombinant virus infectious clone.

The genetic stability of recombinant MDVs is essential for their successful development as vectors. In contrast, if the recombinant virus is developed as a bio-insecticide, evaluation of its stability is a key issue of direct relevance to environmental risk assessment. Although several foreign inserts were integrated and expressed successfully from the MDV vector, the genetic instability of the recombinant MDVs requires further research, as many recombinant virus vectors display genetic instability, as described previously^47^. The genetic stability of each recombinant MDV was confirmed by sequencing. Previous reports suggested that the genetic instability of a recombinant virus is associated with not only the insert size^48^,^49^ but also the G/C content of the insert. Inserts with a G/C content less than 30% seemed to be genetically unstable, regardless of the insert size^49^. Thus, the short insert intronic miRNA expression cassettes and their high G/C contents (approximately 50% G/C contents, see attached sequences) contribute to the genetic stability of the recombinants.

In addition, virus growth curves were plotted to analyse viral proliferation. The growth curves of the C6/36 cells were consistent with those of the w.t. AaeDV. A test of the transmission ability of the recombinant virus from larvae demonstrated that the inserted foreign gene did not affect the proliferation of MDVs. The larval infection ratio, primary infection and site dissemination of the recombinant virus in separated individual mosquitoes were compared with w.t. AaeDV and also did not exhibit any significant differences. These results indicate that the inserted foreign gene inserted had a clear effect on the tested biological characteristics of the MDVs. Interestingly, the recombinant virus induced in larval-rearing water exhibited a significant increase in virus concentration, indicating that viruses are shed by infected larvae and accumulate in the larval environment to facilitate horizontal transmission. The marked increase in virus was observed after 3 d, at which time most of the larvae begin to die (data not shown). Thus, the increase can be explained by the virus released from infected dead mosquito larvae.

In conclusion, the intronic miRNA expression strategy is the first to break the barrier of defective MDVs, in which the non-defective recombinant MDVs display the ability to overexpress and downregulate miRNAs in mosquitoes. This technique not only provides a tool for the functional analysis of mosquito genes but also holds clear commercial applications. Further studies will expand the application of the intronic miRNA expression strategy to the investigation of mosquito biology and paratransgenesis for dengue virus control.

## Additional Information

**How to cite this article**: Liu, P. *et al.* Development of non-defective recombinant densovirus vectors for microRNA delivery in the invasive vector mosquito, *Aedes albopictus. Sci. Rep.* **6**, 20979; doi: 10.1038/srep20979 (2016).

## Supplementary Material

Supplementary Dataset 1

Supplementary Dataset 2

Supplementary Dataset 3

## Figures and Tables

**Table 1:** Impact of recombinant viruses mediated miRNAs over-expression and down-regulation on development of *Aedes albopictus.*

Group	Larval stage span (Days ± SE)	Pupation (% ± SE)	Emergence (% ± SE)
Blank control	5.93 ± 0.10a	0.91 ± 0.01a	0.93 ± 0.02a
AaeDV	6.87 ± 0.25b	0.85 ± 0.02b	0.86 ± 0.03b
VrepUCA-7	7.12 ± 0.21b	0.84 ± 0.01b	0.81 ± 0.02b
VrepUCA-210	6.93 ± 0.09b	0.87 ± 0.02b	0.80 ± 0.03b
VrepUCA-7s	7.32 ± 0.28c	0.81 ± 0.05b	0.81 ± 0.03b
VrepUCA-210s	6.99 ± 0.16b	0.82 ± 0.06b	0.82 ± 0.03b
VrepUCA-941-1s	6.82 ± 0.13b	0.87 ± 0.02b	0.83 ± 0.02b

*The mean values were calculated from 200 larvae per tested virus in triplicates. Means within the same column with different letters are significantly different (P ≤ 0.05).
